# Supplementary material for: Neural dynamics of perceptual inference and its reversal during imagery
Source: eLife. 2020 Jul 20;9:e53588. doi: 10.7554/eLife.53588 (PMC7371419; doi:10.7554/eLife.53588)
Supplement: Supplementary file 1. — (a) Bayesian Information Criterion (BIC) for linear mixed-effects model explaining imagery reactivation time with perception model time. (b) BIC for linear mixed-effects model explaining imagery reactivation time with perception model time after permuting the stimulus-class labels. (c) BIC for linear mixed-effects model explaining imagery reactivation time averaged over trials within subject with perception model time. [file elife-53588-supp1.docx]

| Model | Fixed effects | Random effects | df | BIC^1^ |
| --- | --- | --- | --- | --- |
| 1 | Perception time | - | 3 | 323730.0 |
| 2 | Perception time | Subject | 4 | 323462.4 |
| 3 | Perception time | Subject + Perception time \| Subject | 6 | did not converge |
| Best fit | Perception time | Subject + Trial | 5 | 322911.5 |
| 5 | Perception time | Subject + Perception time \| Subject + Trial + Perception time \| Trial | 9 | did not converge |

**Supplementary File 1a.** Bayesian Information Criterion (BIC) for linear mixed-effects model explaining imagery reactivation time with perception model time.

1. BIC is given in a smaller is better measure

| Model | Fixed effects | Random effects | df | BIC^1^ |
| --- | --- | --- | --- | --- |
| 1 | Perception time | - | 3 | 326310.8 |
| 2 | Perception time | Subject | 4 | 326042.3 |
| 3 | Perception time | Subject + Perception time \| Subject | 6 | did not converge |
| Best fit | Perception time | Subject + Trial | 5 | 325415.1 |
| 5 | Perception time | Subject + Perception time \| Subject + Trial + Perception time \| Trial | 9 | did not converge |

**Supplementary File 1b.** BIC for linear mixed-effects model explaining imagery reactivation time with perception model time after permuting the stimulus-class labels.

1. BIC is given in a smaller is better measure

| Model | Fixed effects | Random effects | df | BIC^1^ |
| --- | --- | --- | --- | --- |
| 1 | Perception time | - | 3 | -724.5 |
| 2 | Perception time | Subject | 4 | -935.9 |
| Best fit | Perception time | Subject + Perception time \| Subject | 6 | -956.7 |

**Supplementary File 1c.** BIC for linear mixed-effects model explaining imagery reactivation time averaged over trials within subject with perception model time.

1. BIC is given in a smaller is better measure
